# Transcriptome analysis of chicken ES, blastodermal and germ cells reveals that chick ES cells are equivalent to mouse ES cells rather than EpiSC

**DOI:** 10.1016/j.scr.2014.11.005

**Published:** 2015-01

**Authors:** Christian Jean, Nidia M.M. Oliveira, Sittipon Intarapat, Aurélie Fuet, Clément Mazoyer, Irene De Almeida, Katherine Trevers, Sharon Boast, Pauline Aubel, Federica Bertocchini, Claudio D. Stern, Bertrand Pain

**Affiliations:** a INSERM, U846, Stem Cell and Brain Research Institute, Bron, France; b INRA, USC1361, Bron, France; c Université de Lyon, Lyon 1, UMR S 846, Lyon, France; d Department of Cell and Developmental Biology, University College London, London, UK

## Abstract

Pluripotent Embryonic Stem cell (ESC) lines can be derived from a variety of sources. Mouse lines derived from the early blastocyst and from primordial germ cells (PGCs) can contribute to all somatic lineages and to the germ line, whereas cells from slightly later embryos (EpiSC) no longer contribute to the germ line. In chick, pluripotent ESCs can be obtained from PGCs and from early blastoderms. Established PGC lines and freshly isolated blastodermal cells (cBC) can contribute to both germinal and somatic lineages but established lines from the former (cESC) can only produce somatic cell types. For this reason, cESCs are often considered to be equivalent to mouse EpiSC. To define these cell types more rigorously, we have performed comparative microarray analysis to describe a transcriptomic profile specific for each cell type. This is validated by real time RT-PCR and *in situ* hybridisation. We find that both cES and cBC cells express classic pluripotency-related genes (including cPOUV/OCT4, NANOG, SOX2/3, KLF2 and SALL4), whereas expression of DAZL, DND1, DDX4 and PIWIL1 defines a molecular signature for germ cells. Surprisingly, contrary to the prevailing view, our results also suggest that cES cells resemble mouse ES cells more closely than mouse EpiSC.

## Highlights

• First comparison of transcriptomic contents of avian stem cells
• Identification of avian pluripotency associated set of genes
• Identification of chicken germ cell specific set of genes
• Establishment of similarities between cES and mES cells
• Evidence for a heterogeneous molecularly status of the early chicken embryos

## Introduction

Embryonic stem (ES) cells were first generated from mouse embryos in 1981 (Evans and Kaufman, 1981; Martin, 1981), then in the primates (Thomson et al., 1995) including human (Thomson et al., 1998). ES and ES-like cells have also been obtained from other mammalian species (Kumar De et al., 2011; Gómez et al., 2010; Hatoya et al., 2006; Verma et al., 2007; Li et al., 2004) and, apart from the rat (Buehr et al., 2008; Li et al., 2008), characterised mainly in short-term culture by the expression of genes associated with pluripotency but without testing for somatic chimaerism or germline transmission.

In non-mammalian species, cell lines have been generated from zebrafish and medaka fish (Hong et al., 2011; Yi et al., 2009; Wakamatsu et al., 1994), some of which are able to contribute to chimaeras and to be transmitted through the germ line. In birds, chicken ES cell lines have been derived from cultures of chicken blastodermal cells (cBC) taken from Stage X-XII (Eyal-Giladi and Kochav, 1966) embryos (Pain et al., 1996; Petitte et al., 2004; Lavial et al., 2007). These cES cells are positive for telomerase activity, alkaline phosphatase and the antigen SSEA1 (Lavial and Pain, 2010) and can contribute to all somatic tissues when injected into recipient embryos (Pain et al., 1996; van de Lavoir et al., 2006a,b) as well as in vitro (Pain et al., 1996; Boast and Stern, 2013). However, in contrast to cBCs, which exhibit a germ line contribution (Carsience et al., 1993) and despite their expression of EMA1, considered as a good germ cell marker (Urven et al., 1988), chicken ES cells have very limited ability to contribute to the germ line (Pain et al., 1996; Petitte et al., 2004). In contrast, long term cultured PGCs are able to colonise the germ line when injected back into recipient embryos. Functional PGCs can be obtained from the embryonic blood of stage 14-17 HH (Hamburger and Hamilton, 1951) embryos (Naito et al., 2004; van de Lavoir et al., 2006a,b; Macdonald et al., 2010, 2012; Park and Han, 2012) or from the gonads of stage 28-30 (Hamburger and Hamilton, 1951) embryos (Ha et al., 2002; Park et al., 2003; Song et al., 2014). These PGCs can be established and maintained in culture using a similar medium as described for cES (Pain et al., 1996), but supplemented with higher concentrations of FGF and SCF and by promoting the non-adherent floating cells that emerge in culture (van de Lavoir et al., 2006a,b; Macdonald et al., 2010). These cells now appear very promising for generating genetically modified chickens (Park and Han, 2012; Macdonald et al., 2012; Schusser et al., 2013).

Further complexity of the ES cell state has now been revealed both with the identification in the mouse of “Epiblast stem cells” (EpiSC) (Tesar et al., 2007; Brons et al., 2007) and with the characterisation of naïve and primed states (Nichols et al., 2009; Marks et al., 2012). At least in the mouse and rat (Chambers and Smith, 2004; Buehr et al., 2008; Li et al., 2008), ES cells have been shown to be LIF dependent but independent from the Erk-MAPK and GSK3 signalling pathways, as demonstrated by the use of specific chemical inhibitors (the so-called “2i” medium; Nichols et al., 2009). Female rodent ES cells possess two active X chromosomes and are able to generate chimaeras with both somatic and germinal contribution when injected into recipient embryos. In contrast, EpiSC are FGF-, Activin- and MEK-dependent, contain an inactive X chromosome and are not transmitted through the germ line (Chenoweth et al., 2010; Han et al., 2010; Zhou et al., 2010). In mouse, ES and EpiSC cell types can be interconverted using either specific small molecules and culture conditions (LIF in 2i medium vs Activin and FGF) or through the overexpression of specific transcription factors such as Klf4, Klf2, Stat3, Nr5a1, Nr5a2 (Guo et al., 2009; Guo and Smith, 2010; Zhou et al., 2010; Bernemann et al., 2011; De Los Angeles et al., 2012). It is still highly debated whether these states can be defined and characterised in other mammalian species, including the human and other primates. Because of the inability of chick ES cells to contribute to the germ line, it has generally been thought that these are more akin to mouse EpiSC than to true mES cells (van de Lavoir et al., 2006a,b; Intarapat and Stern, 2013b).

To help define the chicken ES cells and PGC better, it would be advantageous to study the expression profiles of various genes related to pluripotency and to the germ cell identity, as has been done in mammalian cells (see Pashai et al., 2012). Here we report a comparative microarray analysis using various chicken stem cells. We define a molecular signature for avian pluripotency, which reveals greater similarity between chicken ES cells and their mouse counterpart than with mEpiSCs.

## Materials and methods

### Cells and embryos

Chicken Blastodermal cells (cBC) from JA57 strain were taken from stage IX-XII (Eyal-Giladi and Kochav, 1976) embryos, dissociated and washed extensively in cold PBS, centrifuged and used for RNA isolation or fixed in cold 4% paraformaldehyde in phosphate buffered saline (PBS) for *in situ* hybridisation. The chicken embryonic stem (cES) cells were established, amplified on inactivated STO feeder cells in proliferative medium containing cytokines and growth factors as described (Pain et al., 1996; Lavial et al., 2007). Long term cultured primordial germ cells (PGCs) were derived either from 48h embryonic blood or from embryonic gonads and maintained in the presence FGF, SCF and BRL conditioned medium as described (Macdonald et al., 2010). The non-tumorigenic BM2 monocytic cell line was grown as described (Solari et al., 1996) using DMEM as basal medium instead of BT88 and used as a proliferative progenitor cell. Primary chicken embryonic fibroblasts (CEF) were prepared from 11-12 day old beheaded and eviscerated embryos according to standard protocols (Gandrillon et al., 1987), maintained and homogenised for 3-4 passages before being used as a somatic cell control. All cell types were prepared (in triplicate) for microarray analysis; separate cultures were collected for real-time PCR analysis.

### RNA extraction

RNA was extracted from cell pellets using 1 ml of TRIzol® Reagent (Invitrogen) for 5x10^6^ cells according to the manufacturer’s recommendations. After purification, the isolated RNA was resuspended into RNAase free water and its quantity determined by a Nanodrop-2000 Spectrophotometer (Thermo Scientific) to adjust the final concentration to 1 μg.μl^− 1^. RNA extracted from cBC cells was additionally purified with an affinity column (Qiagen) before being quantified; 500 ng of RNA were loaded on a 1% agarose gel to determine RNA quality.

### RNA labelling and array hybridisation

Following One-Colour Microrray-Based Gene Expression Analysis v6.5 (Agilent Technologies), 200 ng of RNA were Cy3-labeled using the Low Input QuickAmp labelling Kit (Agilent). Cy3 incorporation and final concentration of labelled complementary RNAs (cRNAs) were checked using a Nanodrop-2000 Spectrophotometer. 1.65 μg of Cy3 labelled cRNA was fragmented for 30 minutes using the Gene Expression Hybridisation Kit (Agilent) and loaded on 4× 44k GE chicken V2 slides (Agilent). Assembled chambers were placed in a rotisserie in a hybridisation oven at 65 °C for 17 hours. After hybridisation, the slides were washed according to the manufacturers’ instructions (Agilent) and scanned using a GenePix 4000B microarray scanner (Molecular Devices). The .gal file containing the annotations (version 20120328) was used to grid the slide and the spot intensity extracted with GenePixPro v6.1.0.4 sofware and saved as a .gpr file.

### Raw data analysis

Quantification of expression and identification of differentially expressed genes were done in R v2.10.1 using the Limma 3.2.3 library and single channel analysis. Normalisation within and between arrays were done using Lowess and average quantile methods respectively. Background correction was done using the Normexp method. The average of the intensity was taken for duplicate spots. To identify differentially expressed genes, statistical tests with moderated t-statistic were performed. Multiple comparisons were then corrected by the Benjamini and Hochberg false discovery rate method and a corrected p-value of < 0.05 was chosen as significant. Significant lists of genes were then filtered with a threshold of Log_2_ of the Fold change (Log_2_ FC) > 2.

### Clustering analysis

For each file obtained per cell type, the list of differentially expressed genes was analysed using the eVenn package (version 2.1.6) to generate Venn diagrams and the common differentially expressed genes between all the tested cell types. The online David Bioinformatics Resource 6.7 was used to define clusters of genes based on predicted functions. The R package FactoMineR was used to perform PCA on all 44k probes. For heatmap clustering and virtual Array, GSE38168 and GSE17984, GSE43398, GSE33953 from the NCBI GEO datasets (http://www.ncbi.nlm.nih.gov/geo/) provided data for the chicken and mouse respectively and were integrated in the comparison as described (Heider and Alt, 2013). Anova analysis was performed on the 11708 common gene names found for the chicken arrays and on the 8749 found once the mouse ES and EpiSC data are integrated. The array data are available through the GSE 61221 dataset.

### qPCR

After DNAse treatment (Invitrogen), 1 μg of total RNA was reverse-transcribed using High Capacity RNA-to-cDNA Master Mix (Applied Biosystems). The cDNA samples were diluted 5-fold for qPCR analysis using StepOnePlus^TM^ Real-Time PCR System (Applied BioSystems). Each cDNA sample was amplified in triplicate, each well containing 1 μl of diluted cDNA, 300 nM of primers and Fast SYBR® Green Master Mix 1x Final (Applied Biosystems). Using the comparative ΔCt method, the StepOne Plus TM software provides the RQ value for each sample with the RSP17 as internal control as described (Lavial et al., 2007, 2009). Primers are listed on Table S1.

After DNAse treatment (Invitrogen), 1 μg of total RNA was reverse-transcribed using High Capacity RNA-to-cDNA Master Mix (Applied Biosystems). The cDNA samples were diluted 5-fold for qPCR analysis using StepOnePlus^TM^ Real-Time PCR System (Applied BioSystems). Each cDNA sample was amplified in triplicate, each well containing 1 μl of diluted cDNA, 300 nM of primers and Fast SYBR® Green Master Mix 1x Final (Applied Biosystems). Using the comparative ΔCt method, the StepOne Plus TM software provides the RQ value for each sample with the RSP17 as internal control as described (Lavial et al., 2007, 2009). Primers are listed on Table S1.

### In situ hybridisation of embryos and cultures

Whole-mount *in situ* hybridisation was carried out in chick embryos at various stages of development as previously described (Streit and Stern, 2001). Digoxigenin-labeled probes (Table S3) were synthesised from pBlueScript or pGEMT-easy plasmids using T3, T7 or SP6 RNA polymerase as appropriate. After hybridisation, embryos were fixed in 4% paraformaldehyde in PBS, washed in PBS, and photographed in whole-mount under either a Leica M10 or an Olympus SZH10 dissecting microscope. The same procedure was used for studying gene expression in whole gonads dissected from embryos at stage 25 (indifferent stage) and stage 35 (following sexual differentiation). To examine gene expression in cultured cells, the above in situ hybridisation protocol was adapted to adherent cells in 24 well plates (300 μl of each solution used per well). The procedure was almost identical to that used for whole embryos except that no Proteinase-K treatment was used.

## Results

### Identification of differentially expressed genes between chicken stem cells and fibroblasts

**Figure 1:**
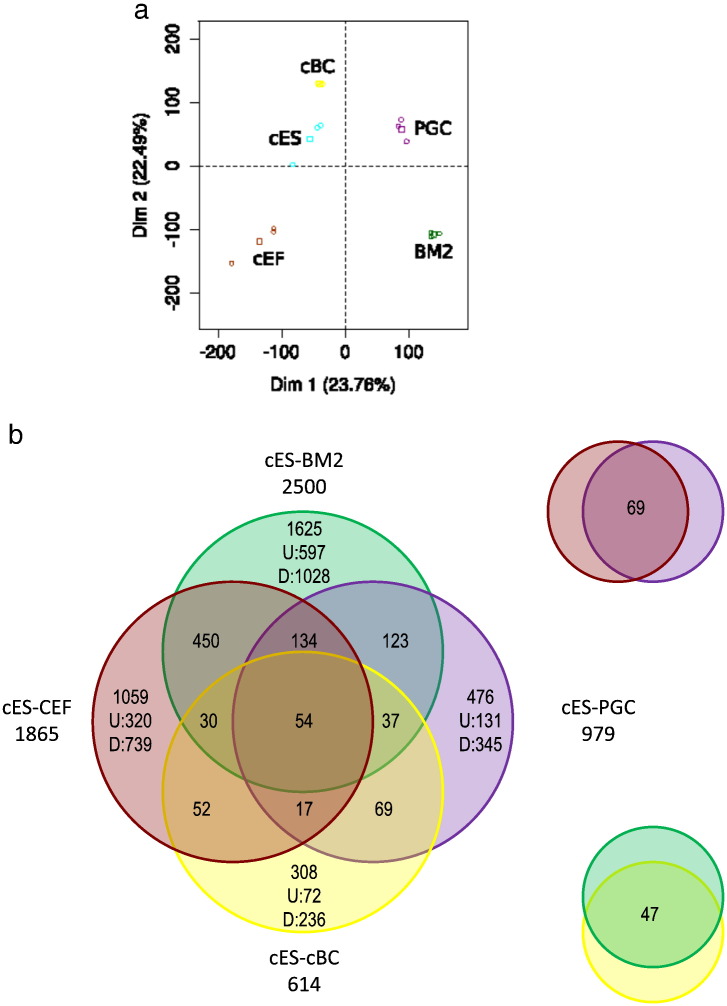
The cells analysed are molecularly different. a: PCA analysis, calculated on all probe sets, demonstrates that chicken embryonic fibroblasts (CEF), monocytic BM2 progenitor cells, chicken embryonic stem (cES) cells, primordial germ cells (PGC) and blastodermal cells from stage X (EG & K) chick embryos (cBC) are molecularly different cell types as none of them overlap either on the first or second axis. Each sample (circle) is plotted as well as the barycentre of the triplicate (square). The same color code was used all along the manuscript to help for a simple comparison between the different cell types. b: The Venn diagram presents genes that are differentially expressed between the tested cell types, including CEF, the monocytic BM2 progenitor cell, cES cells, PGC and cBC.

The transcriptomic landscape between different chicken stem cells and fibroblasts was established using the most recent version of commercially annotated microarrays. For each cell type, three independent samples were hybridised and a first PCA analysis obtained from all probe sets demonstrates the molecular differences between the cell types analysed (Fig. 1A). Using cutoffs of p-value ≤ 0.05 and Log_2_(FC) ≥ 2, genes presenting at least one difference in expression between the different cell types were selected (Table S2). The genes were classified as either up-regulated (U), down-regulated (D) or not differentially expressed (n) between the different cells. A total of 5944 differentially expressed unique identifiers (target IDs) were defined for CEF, 5942 for BM2 cells, 4550 for cES, 5465 for PGC and 5522 for cBC.

The transcriptomic landscape between different chicken stem cells and fibroblasts was established using the most recent version of commercially annotated microarrays. For each cell type, three independent samples were hybridised and a first PCA analysis obtained from all probe sets demonstrates the molecular differences between the cell types analysed (Fig. 1a). Using cutoffs of p-value ≤ 0.05 and Log_2_(FC) ≥ 2, genes presenting at least one difference in expression between the different cell types were selected (Table S2). The genes were classified as either up-regulated (U), down-regulated (D) or not differentially expressed (n) between the different cells. A total of 5944 differentially expressed unique identifiers (target IDs) were defined for CEF, 5942 for BM2 cells, 4550 for cES, 5465 for PGC and 5522 for cBC.

The Venn diagrams (Figs. 1b and S1) illustrate comparisons between the genes expressed in different cell types. The number of differentially expressed genes is higher between somatic fibroblasts (CEF) and all the stem cells (cES, cBC & PGC) than between the latter three (non-somatic) cell types (Table 1). This difference is even more pronounced with the monocytic BM2 cell line, probably reflecting the fact that this cell type is engaged in a very specific diffentiation pathway. In addition, the use of CEF as somatic cells allows removal of many housekeeping genes common to all dividing cell types, leading to a better comparison of genes specific for each stem cell type. Interestingly, there are more differences between the PGC and either cES or cBC (with 979 and 1204 differentially target ID genes, respectively) than between cES and cBC, which only differ by 604 genes.

As the chicken genome annotation is still incomplete, many of the genes that are detected to be differentially expressed still lack gene IDs. In this study, an average of only 57.8% (ranging from 58.2, 57.1, 57.8, 57.1 and 58.7 for CEF, BM2, cES, PGC and cBC respectively) of differentially expressed genes can be associated unambiguously with a gene symbol. Non-annotated genes were not considered, and we did not look for likely mammalian homologues. However their identifiers were kept in all the tables for future analysis as annotation of the chicken genome improves.

Generally, the two somatic cell types (fibroblasts and BM2 cells) have a greater number of repressed genes than genes whose expression increases relative to the three embryonic and stem cell types, with an average of two to three times more down- than up-regulated. Specifically, 511 and 666 probes (corresponding to 294 and 388 annotated genes, respectively) are found to be up-regulated for CEF and BM2 respectively against all the other cell types. In contrast, only 37, 110 and 182 probes (15, 59 and 71 annotated genes) are specifically expressed in the cES, cBC and PGC cells. The 15 cES specific genes are: LOC770611, UPK3BL, PERP, CLDN3, LOC415324, PPL, SYT8, ENPP2, C9H2orf82, GALNT3, PRR13, GPR87, LOC424593, FGF1 and GOLPH3LPPL. The top 15 cBC genes are CCNA1, HEMGN, EOMES, DKK1, HSP25, RNF152, SOX7, FGF8, SOCS1, NR0B1, LOC768589, GATA5, NOG, GRP, RBP4. The top 15 PGC specific genes are DAZL, ENS-3, CTNNA3, LOC421805, ELAVL4, DND1, TTC39A, GTSF1, CALR3, DDX4, GCNT2, PIWIL1, GPR149, FEZF2, PNLDC1. The full list of genes is presented on Table S3-1.

### A “pluripotency signature”

A Log_2_(FC) value was obtained for each gene found to be differentially expressed in at least one cell type. For one cell type, a maximum of four values of Log_2_(FC) can be obtained (one against each other cell type where differential expression is seen); by adding them, a ‘FC score’ can be calculated for each gene. The value of this score is shown on the gene list for each cell type and ranges from + 32.4 to -26.9 (for CEF), from + 40.9 to -33.2 (for BM2), from + 27.6 to − 19.6 (for cES), from + 24.4 to − 19.8 (for cBC) and from + 30.7 to − 25.4 (for PGC) (Table S2). The highest positive numbers reflect the most specific genes expressed in a given cell type and conversely, the lowest negative numbers indicate the least expressed genes for the cell type. Using this FC score, the BM2 cells stand out as having the highest degree of difference between all the compared cell types, reflecting a completely differentiated cell type.

A Log_2_(FC) value was obtained for each gene found to be differentially expressed in at least one cell type. For one cell type, a maximum of four values of Log_2_(FC) can be obtained (one against each other cell type where differential expression is seen); by adding them, a ‘FC score’ can be calculated for each gene. The value of this score is shown on the gene list for each cell type and ranges from + 32.4 to -26.9 (for CEF), from + 40.9 to -33.2 (for BM2), from + 27.6 to − 19.6 (for cES), from + 24.4 to − 19.8 (for cBC) and from + 30.7 to − 25.4 (for PGC) (Table S2). The highest positive numbers reflect the most specific genes expressed in a given cell type and conversely, the lowest negative numbers indicate the least expressed genes for the cell type. Using this FC score, the BM2 cells stand out as having the highest degree of difference between all the compared cell types, reflecting a completely differentiated cell type.

To establish a chicken ‘stem cell’ signature, we used this FC score to identify the genes that are most significantly expressed in cES, cBC and PGCs, compared to CEF and BM2 cells. The level of + or8 (Log_2_(FC) ≥ 2.0 in comparisons with each of 4 other cell types) was chosen to indicate genes more specific to one molecular signature. On an initial list of 536 and 702 target IDs for CEF and BM2, with 344 and 402 gene IDs respectively presenting a FC score superior to − 8, 88 genes were selected to be commonly expressed in cES, cBC and PGC cells (Table S3-1). Analyzing this list using the David software, 82 genes were retained; several GO terms are enriched in this list: developmental process, regulation of biological process, regulation of gene expression, intracellular organelle and nucleus, nucleic acid binding and transcription regulator activity are particularly represented in pluripotent cells (cES and cBC) as compared to the two somatic cell types. One of the larger sets, sharing the GO term ‘Regulation of transcription’, comprises 17 genes, predominantly encoding transcription factors: CNOT2, DNMT3B, EAF2, IRF6, KLF1, NANOG, SOX3, CDX2, ID2, LIN28, NR6A1, OTX2, SALL1, SALL4, ACTR2B, TESC and TFAP2A. cPOUV/OCT4 (not recognised by the David software through its chicken name, POUV) should be added to this list. Genes encoding RNA binding proteins are also present including ZARL1, LIN28 and IGF2BP3. We can also identify several other clusters such as growth factors and cytokines involved in specific signalling pathways including CFC1B, DACT2, WNT5A, FGF12, CBLN2, WNT3A and receptors including ERBB3, ACVR2B, FGFR2, CMTM8. Small clusters of genes are linked to cell structural features such as Cell adhesion molecules (CAMs) like EPCAM, intermediate filaments (KRT8, KRT19 and NEFM), cell adhesion and junctions (CLDN1, DSP, CGNL1, GJB6) transmembrane and matrix-bound proteins (MPZL3, TSPAN13, CD24, CDH1, LAMA1, TMOD1). Solute carriers and channels (SLC45A2, ANO3, SCNN1A, SLC16A9, SLC34A2) are also present with enzymes involved in specific amino acid, sugar or lipid metabolism (ARG2, GCNT2, GLUL, CDS1, SDSL GPAT2). A few other genes are also noteworthy including TRIM71, a E3 ubiquitin ligase, H1FOO, a unique histone found in the germinal lineage and ERNI/ENS1, a protein with roles in pluripotency and early neural development (also not recognised by David). A summary is presented in Table S3-2.

**Figure 2:**
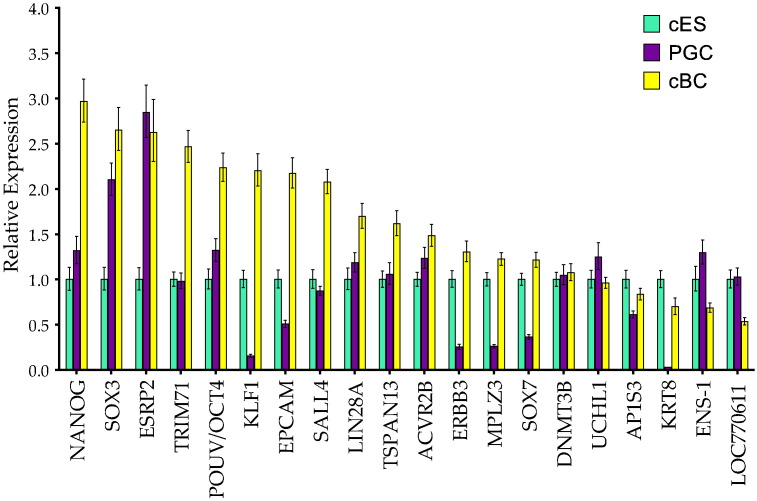
Pluripotency-associated genes are expressed in the different tested stem cell subtypes. The expression of pluripotency-related genes was analysed in the three cell types (cES, PGCs and cBC) by real time RT-PCR. Expression of NANOG, SOX3, ESRP2, TRIM71, POUV/OCT4, KLF2/KLF1, SALL4, LIN28A, TSPAN13, ACVR2B, ERBB3, MPLZ3, SOX7, DNMT3B, UCHL1, AP1S3, KRT8, ENS-1, LOC660611, is in the same range of expression for cES and cBC (from 23.0 to 0.5 fold), with more various expression level for the PGCs (from 2.8 to 0.15 – for KLF2). Expression was taken to be 1 in cES as a reference, and two independent samples were run, each in triplicate. Error bars indicate SD.

**Figure 3:**
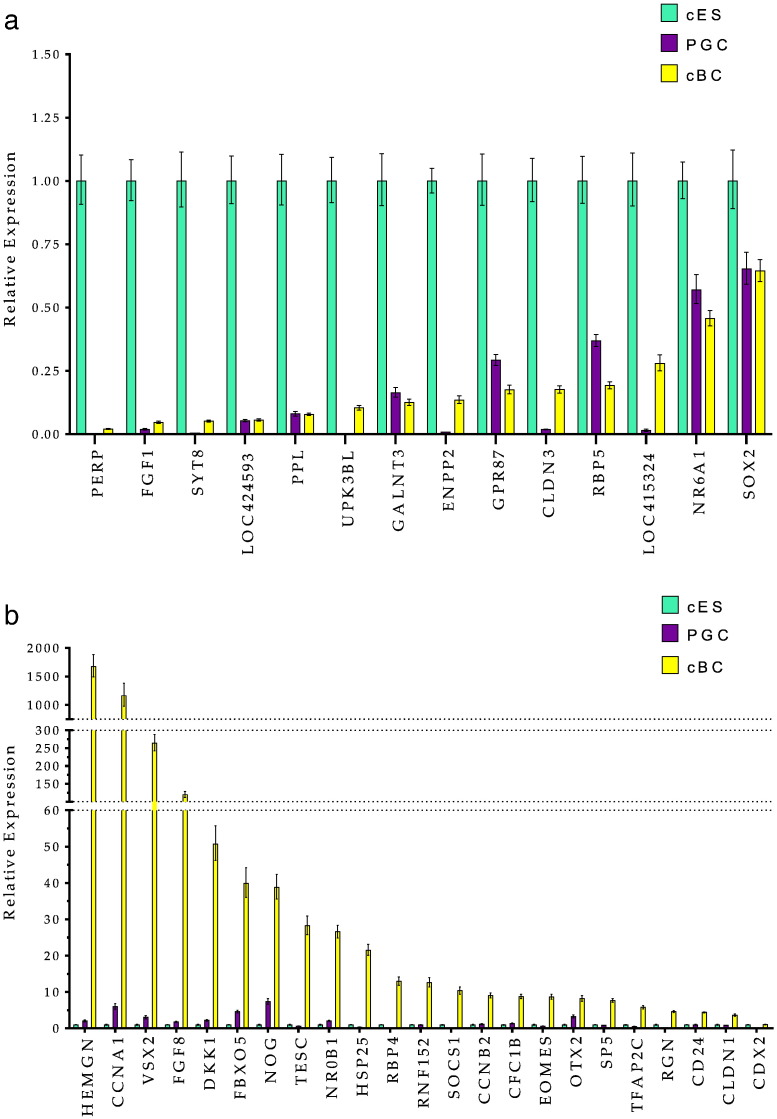
Differential gene expression in different stem cell subtypes. a: Expression of PERP, FGF1, SYT8, LOC424593, PPL, UPK3BL, GALNT3, ENPP2, CLDN3, RBP5, CLDN3, and NR6A1 and SOX2 genes was analysed by real time RT-PCR in cES, PGC and cBC. Expression was taken to be 1 in cES as a reference and two independent samples were run, each in triplicate. Error bars indicate SD. b: Expression of HEMGN, CCNA1, VSX2, FGF8, DKK1, FBXO5, NOG, TESC, NR0B1, HSP25, RBP4, RNF152, SOCS1, CCNB2, CFC1B, EOMES, OTX2, SP5, TFAP2C, RGN, CD24, CLDN1 and CDX2 genes was analysed by real time RT-PCR in cES, PGC and cBC. These genes appear to be more specific to cBC cells with a lower expression in either cES or PGC. Expression was taken to be 1 in cES as a reference and two independent samples were run, each in triplicate Error bars indicate SD.

Real time RT PCR analysis of pluripotency-related genes reveals the expected profile for almost all of the main transcription factors including LIN28A, KLF1, NANOG, cPOUV/OCT4, SALL4, SOX3, with relative expression ranging from 3 to 1 for cBC when compared to cES (Fig. 2). This small cluster could represent a typical pluripotency-related chicken gene set. SOX3 and NANOG are expressed slightly more strongly in cBC compared to cES, whereas SOX2 gene is almost not expressed in cBC cells. The other genes tested such as ESRP2, TRIM71, EPCAM, TSPAN13, are also expressed slighty more in cBC than in cES. As illustrated on Fig. 3a, the genes PERP, FGF1, SYT8, LOC424593, PPL, UPK3BL, GALNT3, ENPP2, GPR87, CLDN3, RBP5, LOC415324, NR6A1 and SOX2 are expressed more strongly in cES and define a more specific signature for this cell type. Likewise, expression of CCNA1, HEMGN, CCNA1, VSX2, FGF8, DKK1, FBXO5, NOG, TESC, NR0B1, HSP25, RBP4, RNF152, SOCS1, CCNB2, CFC1B, EOMES, OTX2, SP5, TFAP2C, RGN, CD24, CLDN1 and CDX2 genes defines the signature of blastoderm cells freshly obtained from the embryo (Fig. 3b).

### A germ cell signature

**Figure 4:**
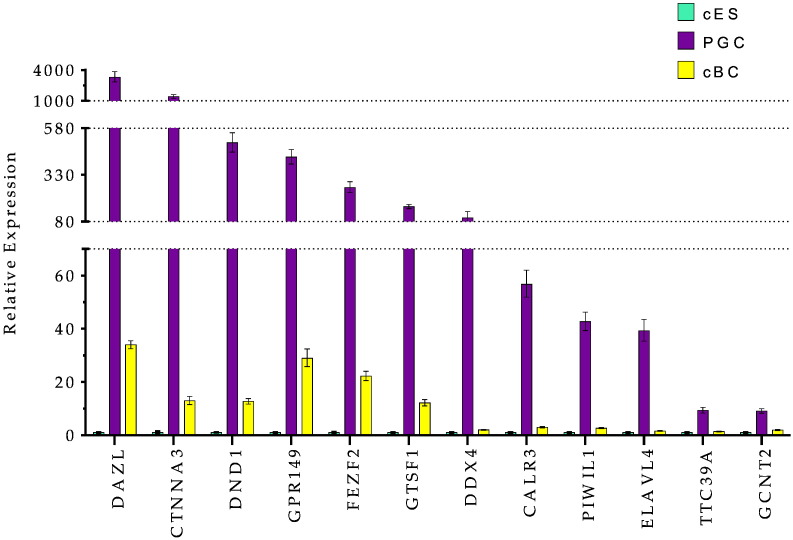
Specific expression of Germ cell related genes in PGCs. Expression of DAZL, CTNNA3, DND1, GPR149, FEZF2, GTSF1, DDX4, CALR3, PIWIL1, TTC39A, GCNT2genes was analysed by real time RT-PCR in cES, PGC and cBC. These genes appear to be more specific to the PGCs with a low expression in cES cells and a variable expression in cBC reflecting the presence of already individualised germ cells at this stage of development. Expression was taken to be 1 in cES as a reference and two independent samples were run, each in triplicate. Error bars indicate SD.

Using the same approach, the highest FC-score for PGCs (from + 30 to + 8) (Table S2-PGC) defines a list of genes including DAZL, ELAVL4, DND1, GTSF1, DDX4, PIWIL1, FKBP6, ZAR1, TSPAN1, CTNNA2, some genes with a Tudor domain (TDRD5, TDRD9, TDRKH) and genes reported to be important for the germinal lineage such as SPAG4, DMRT1, SCYP3, MAEL, KIT and STRA8 (Fig. S2). By real time PCR, the expression of DAZL, CTNNA3, DND1, GPR149, FEZF2, GTSF1, DDX4, CALR3, PIWIL1, ELAVL4, TTC39A and GCNT2 is more specifically associated with the germinal lineage (Fig. 4). Some of these genes are also detectable in cBC compared to cES, likely to reflect the presence of germ cells at this blastoderm stage of development, as previously reported (Tsunekawa et al., 2000). In both pluripotent and germ line analysis, the level of expression of the genes analysed by real time PCR (Figs. 2, 3A, B and 4) is almost undetectable in both CEF and BM2 cells with the exception of HEMGN, GPR149, FBXO5, SOCS1 andGPR87, for which the expression in BM2 is variable (Fig. S3).

Using the same approach, the highest FC-score for PGCs (from + 30 to + 8) (Table S2-PGC) defines a list of genes including DAZL, ELAVL4, DND1, GTSF1, DDX4, PIWIL1, FKBP6, ZAR1, TSPAN1, CTNNA2, some genes with a Tudor domain (TDRD5, TDRD9, TDRKH) and genes reported to be important for the germinal lineage such as SPAG4, DMRT1, SCYP3, MAEL, KIT and STRA8 (Fig. S2). By real time PCR, the expression of DAZL, CTNNA3, DND1, GPR149, FEZF2, GTSF1, DDX4, CALR3, PIWIL1, ELAVL4, TTC39A and GCNT2 is more specifically associated with the germinal lineage (Fig. 4). Some of these genes are also detectable in cBC compared to cES, likely to reflect the presence of germ cells at this blastoderm stage of development, as previously reported (Tsunekawa et al., 2000). In both pluripotent and germ line analysis, the level of expression of the genes analysed by real time PCR (Figs. 2, 3a, b and 4) is almost undetectable in both CEF and BM2 cells with the exception of HEMGN, GPR149, FBXO5, SOCS1 andGPR87, for which the expression in BM2 is variable (Fig. S3).

### Chicken ES cells cluster with mES cells

**Figure 5:**
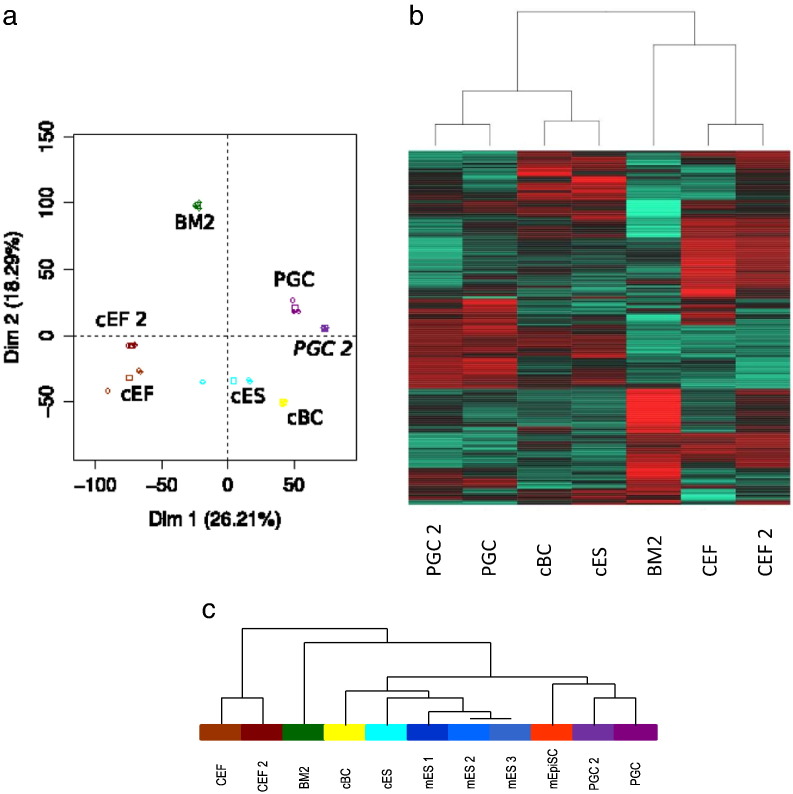
PCA and heatmap analysis reveals clustering of CEF and PGC with previous data, and cES with cBC (a, b) and with mES in an virtual Array (c). a: PCA analysis was performed on the 11708 common genes between the data from this study (GSE61221) (PGC, cBC, cES, BM2 and CEF) and those provided by the PGC and CEF samples (PGC 2, CEF 2) of the GSE38168 microarray data as described in Materials and Methods. CEF and PGC from the two studies clustered together. Each sample (circle) is plotted as well as the barycentre of the triplicate (square). b: The heatmap analysis was performed by  ANOVA analysis of the 11708 common genes between the data from this study (GSE61221) and those of the GSE38168 set as listed in A. The heatmap presented was performed with the first 1000 ranked genes. c:. By incorporating the mouse ES and EpiSC cells in a virtual array approach as described in Materials and Methods, a clustering tree reveals that cES cells cluster more closely with mES cells than with EpiSC, whereas the latter are more closely related to PGC.

Based on Anova analysis of the 11708 common genes between these data and those from a previous analysis of CEF and PGC (Rengaraj et al., 2012), PCA analysis reveals the good agreement found between the CEF and PGC cells from the two analyses and a heat map of the first 1000 most discriminating genes reveals a clear relationship between cES and cBC (Fig. 5b). By incorporating mouse NCBI GEO data sets (see Materials and Methods), Anova analysis of 8759 common genes between the mouse and the chicken data demonstrates that the cES cluster with mES cells rather than with EpiSC (Fig. 5c). Moreover, when looking at the expression profiles of chicken homologues of mouse genes identified to be differentially expressed between mES and mEpiSC (Bao et al., 2009; Tesar et al., 2007), most of are found to be expressed in our microarray analysis either in cES or in cBC, or in both. This includes DAZL, NR0B1, PIWI as the best discriminators between the two states. However, genes with higher expression in EpiSC such as OTX2, CDX2, EOMES, DKK1 and SOX7 are also detected in cES, even if they are more strongly expressed in cBC cells (Figs. 2, 3A and B).

### In situ hybridisation profile

**Fig. 6:**
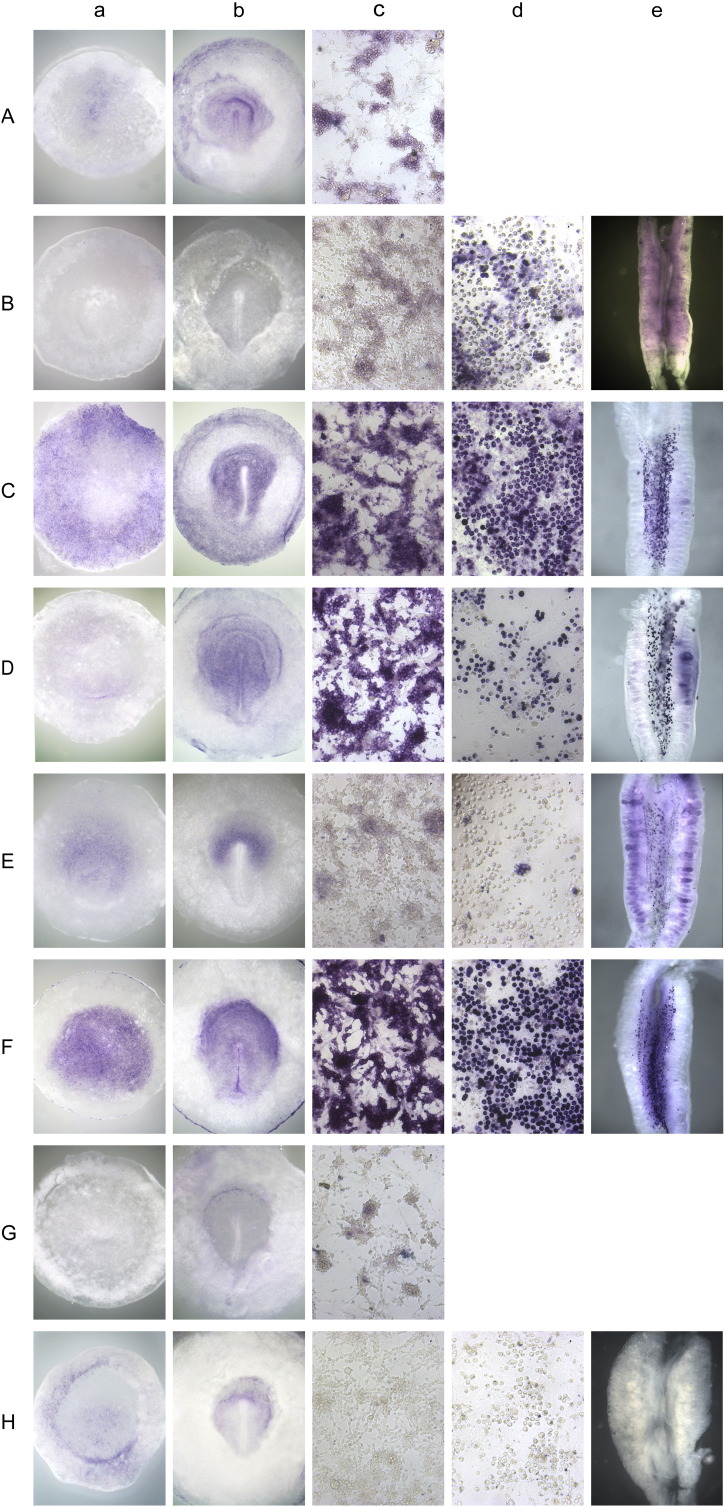

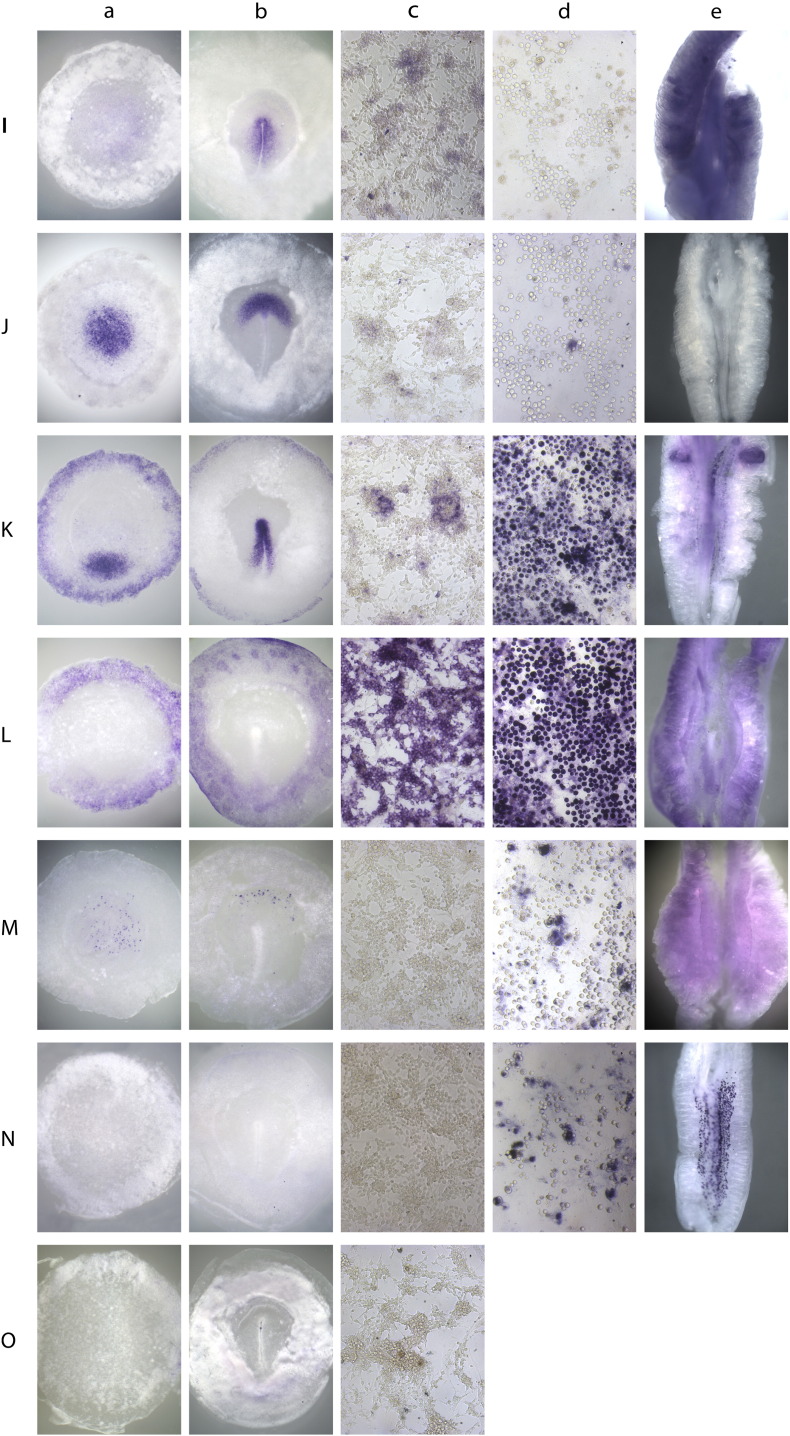
In situ hybridisation of pluripotency-related genes reveals heterogeneous expression in cES and PGC cells in early embryos. A-H: In situ hybridisation for TRIM71 (A), KLF2 (B), NANOG (C), OCT4/POUV (D), SOX3 (E), ENS1/ERNI (F), LOC770611 (G), SOX7 (H), CFCB1 (I), OTX2 (J), EOMES (K), CDX2 (L), DAZL (M), DDX4 N), GTSF1 (O) was performed on pre-primitive-streak stage embryos (a), HH3 +/4 (primitive streak stage) embryos (b), cES (c), PGC (d) and 7 day old embryonic gonads (e) as indicated in Materials and Methods. Expression of NANOG (C), POUV/OCT4 (D), ENS1/ERNI (F) and CDX2 (L) is detected homogeneously in both cES and PGC cells. The other genes present a heterogeneous profile for cES and PGC cells.

To investigate whether cells express the most important markers uniformly or whether there is cell heterogeneity, we used *in situ* hybridisation on *in vitro* cultured cells (both cES and PGCs) and in early embryos. First, in agreement with the PCR analysis (Fig. 3b), the pluripotency-related genes TRIM71, NANOG, cPOUV/OCT4, SOX3 and ENS-1/ERNI are found to be expressed in early pre-primitive-streak stage embryos (Figs. 6Aa, Ca, Da, Ea, Fa), in cES (**Ab**, **Cb**, **Db**, **Eb**, **Fb**) and in PGCs (**Ac**, **Cc**, **Dc**, **Ec**, **Fc**) in contrast to KLF2, which is barely detected in early embryos (**Ba**) but expressed in cES (**Bb**) and strongly in PGCs (**Bc**). The expression profiles of LOC660611 (Figs. 6Ga-c) and SOX17 (Figs. 6Ha-c) also reflect the PCR result whereas CFC1B, OTX2, EOMES and CDX2 present strong expression in pre-streak embryos (Figs. 6Ia-Lc). Genes defining a germ cell signature, DAZL, DDX4 and GTSF1, are detected by in situ hybridisation in individual PGCs both in embryos (Figs. 6Ma, Na, Oa) and in cultured PGC lines (Figs. 6Mc, Nc, Oc), but not in cultured cES (Figs. 6Mc, Nc, Oc). The situation for EOMES and CDX2 appears to be more complex as those genes are expressed in cES cells and cultured PGC but not detected in developing gonads.

## Discussion

### A chicken pluripotency associated set of genes

A pluripotent cell is defined as having the ability to contribute to many, or even all, cell types. In rodents, their fates include the germ line. Pluripotency can be demonstrated *in vivo*, when cells are introduced into a recipient embryo, and *in vitro*, when cells differentiate into derivatives of the three primary embryonic cell layers. In chicken, both cBC and cES cells present features of pluripotency. The presence of a lower number of differentially expressed genes between cBC and cES cells when compared to the other stem cell types suggests that the two cell types share a common molecular signature but that the *in vitro* culture process generates a new cell type, cES cells, distinct from the parent cBC. Very few genes are specific to the cES cells alone. Our analysis defines a chicken set of pluripotency genes that is almost coincident with mammalian pluripotency-associated genes, including OCT4, SOX2/3, NANOG, SALL1/SALL4, LIN28 and a KLF family member. OCT4 and NANOG have been identified previously as key actors of the maintenance of cES (Lavial et al., 2007). SALL4 and SALL1, known as major regulators of pluripotency in the mouse (Zhang et al., 2006; Yang et al., 2010; Ng and Surani, 2011) also appear to be strongly expressed in chicken stem cells. One of the most striking differences between mouse and chicken is the expression profile of SOX2/SOX3. SOX2 is almost only found in cultured cES while SOX3 is present in cES, PGC and cBC and detected by *in situ* in early embryos. This expression profile is consistent with previous reports of the expression of this gene family in early embryos (Uwanogho et al., 1995; Rex et al., 1997; Acloque et al., 2011) and in chick ES cells (Lavial et al., 2007), but differs from the mouse, where SOX3 is not expressed in early embryos whereas SOX2 is present in both pre-primitive-streak stage embryos and mES cells (Wood and Episkopou, 1999; Avilion et al., 2003; Masui et al., 2007). The ENS1/ERNI gene, previously identified in cES cells (Acloque et al., 2001) and in early embryos (Streit et al., 2000) but unique to the Gallinacea, is strongly expressed in cES, cBC and PGC and also found by *in situ* hybridisation in both early embryos and embryonic gonads, reminiscent of the expression profiles of OCT4/POUV, NANOG and SOX3 (see also Intarapat and Stern, 2013a).

### A chicken germ cell specific signature

In addition to the pluripotency-related genes, PGC also express specific genes including the well-known markers DAZL, DND1, DDX4 and PIWIL1 as well as GTSF1, CALR3 and GPR149, previously identified to play key roles during mammalian germ cell differentiation (Yoshimura et al., 2009; Ikawa et al., 2011; Edson et al., 2010). LIN28, encoding an RNA binding protein and involved in miRNA processing is also expressed in PGC and was demonstrated to play a role in controlling germ cell development (West et al., 2009). In human cells, LIN28A interacts with ZARL1, found to be strongly expressed in cBC and PGC. ZARL1 was previously identified in chicken oocytes, ovaries and testes and during late embryogenesis, co-localises with PIWIL1 in P-bodies (Michailidis et al., 2010). In contrast, the related gene ZAR1 (Hu et al., 2010) is only detected in cES cells. Together, these genes could be involved in controlling germinal fate.

### Few genes are expressed homogeneously.

The Stage X (EG& K) blastoderm contains some 40,000–50,000 cells. The data obtained through global transcriptomic analysis reflect only the average expression of the genes in the whole tissue, and does not reveal restricted expression to specific cell subtypes, including putative germ cells. In contrast, *in situ* hybridisation allows us to determine whether individual genes are expressed uniformly or in subsets of cells. *In situ* hybridisation on cES and PGC reveals some of the genes to be widely and almost uniformly expressed (OCT4/POUV, NANOG, ENS/ERNI, CDX2), whereas others are only detected in a variable proportion of cells, such as TRIM71, KLF1, SOX3, CFC1B, OTX2 and EOMES for cES cells and KLF2, DAZL and DDX4 for PGCs. This observation suggests that not all cultured cells have the same state and is reminiscent of the finding of a mosaic of epiblast cells expressing the antigen HNK1 at pre-primitive-streak stages (Canning and Stern, 1988), which marks cells destined for the mesendodermal lineage (Stern and Canning, 1990). Either there is stable heterogeneity among cells (which could impact on their ability to contribute to somatic and/or germ lineages), or the expression of these genes is dynamic, or both. Additional reporters would help to define the presence of different subpopulations in the cultured cells as has been done for mouse mES cells (Toyooka et al., 2008).

### cES cells are similar to mES cells

Global comparison of the molecular signature of cES and mES cells leads to clustering of these cell types, reinforcing the view of the ES-cell-like nature of the cultured chicken cells. In contrast, at the molecular level, the chicken cultured PGCs are more similar to mEpiSC, consistent with the close relationship observed in the mouse between EpiSCs and germ cells (Gillich et al., 2012; Hayashi and Surani, 2009).

In conclusion, cES, PGC and cBC cells share a great number of expressed genes including some of the best known pluripotency-associated markers such as POUV/OCT4, NANOG, SOX2/3, KLF2, SALL4 and LIN28. Interestingly at the transcriptome level, the culture process maintains a close relationship between the cBC and their in vitro derivatives, cES cells. cES cells share a highly similar molecular signature with mES cells, apart from some differences for a few specific genes such as EOMES and CDX2, which are strongly expressed in chicken cES cells. As cES cells exhibit some of the specific markers of mammalian ”naïve” cells, we propose that specific culture conditions could be found to obtain and maintain such cells in vitro with the full associated developmental and functional properties of naïve cells, including contribution to the germinal lineage.

The following are the supplementary data related to this article.

Table S1 List of oligonucleotides used in real time RT PCR analysis and for in situ probes synthesis.

Table S2 List of all differentially expressed genes.
The table is composed of the different files of the differentially expressed genes of CEF, BM2, cES, cBC and PGC. The cell type score is indicated (column Q) and calculated by summing all the scores for a defined probe ID.

Table S3-1. Short list of the most specific genes for each cell type
The table presents the top genes according to the FC score as defined.
Table S3-2. Summary of the David Analysis.
The pluripotent associated genes were submitted to the David software.

Fig. S1. Venn diagram of the differentially expressed genes.
The Venn diagram presents the genes that are differentially expressed between the tested cell types, including as a reference the Chicken Embryonic Fibroblasts (CEF) (S1A), the monocytic BM2 progenitor cell (S1B), Primordial germ cells (PGC) (S1C) and Chicken blastodermal cells (cBC) from stage X (EG & K) chick embryos (S1D). Fig. 1 illustrates the Venn diagram by taking the Chicken Embryonic Stem (cES) cells as a reference.
Fig. S2. The PGC score.
The PGC score illustrates the differentially expressed between the PGCs and all the other cell types. This Score is obtained by summing the different Log Fold Change (FC) directly get from the microarray analysis. The higher the score, the more specific are the differentially expressed genes for the cell type. Some genes are listed all along the curve and can be found on Table S2-PGC.
The PGC score illustrates the differentially expressed between the PGCs and all the other cell types. This Score is obtained by summing the different Log Fold Change (FC) directly get from the microarray analysis. The higher the score, the more specific are the differentially expressed genes for the cell type. Some genes are listed all along the curve and can be found on Table S2-PGC.
Fig. S3. Few tested genes are expressed in the different tested stem cell subtypes.
The expression of HEMGN, GPR149, FBXO5, SOCS1 and GPR86 genes was analysed by real time RT-PCR in CEF, BM2, cES, PGC and cBC. Their expression is taken at 1 in cES. These genes are the only ones among those tested in which a significant expression was detected in BM2 cells. SOCS1 is also slightly expressed in CEF. Each sample was run in triplicates.

Supplementary data to this article can be found online at http://dx.doi.org/10.1016/j.scr.2014.11.005.

## Figures and Tables

**Table 1:** 

	Reference cell type				
Differentially target ID expressed	CEF (5944)	BM2 (5942)	cES (4550)	PGC (5465)	cBC (5522)
CEF	X				
BM2	2932	X			
cES	1865	2500	X		
PGC	2770	2880	979	X	
cBC	2865	3066	614	1204	X

## References

[bb0010] Acloque H., Risson V., Birot A.M., Kunita R., Pain B., Samarut J. (2001). Identification of a new gene family specifically expressed in chicken embryonic stem cells and early embryo. Mech. Dev..

[bb0005] Acloque H., Ocaña O.H., Matheu A., Rizzoti K., Wise C., Lovell-Badge R., Nieto M.A. (2011). Reciprocal repression between Sox3 and snail transcription factors defines embryonic territories at gastrulation. Dev. Cell.

[bb0015] Avilion A.A., Nicolis S.K., Pevny L.H., Perez L., Vivian N., Lovell-Badge R. (2003). Multipotent cell lineages in early mouse development depend on SOX2 function. Genes Dev..

[bb0020] Bao S., Tang F., Li X., Hayashi K., Gillich A., Lao K., Surani M.A. (2009). Epigenetic reversion of post-implantation epiblast to pluripotent embryonic stem cells. Nature.

[bb0025] Bernemann C., Greber B., Ko K., Sterneckert J., Han D.W., Araúzo-Bravo M.J., Schöler H.R. (2011). Distinct developmental ground states of epiblast stem cell lines determine different pluripotency features. Stem Cells.

[bb0030] Boast S., Stern C.D. (2013). Simple methods for generating neural, bone and endodermal cell types from chick embryonic stem cells. Stem Cell Res..

[bb0035] Brons I.G., Smithers L.E., Trotter M.W., Rugg-Gunn P., Sun B., de Sousa Chuva, Lopes S.M., Howlett S.K., Clarkson A., Ahrlund-Richter L., Pedersen R.A., Vallier L. (2007). Derivation of pluripotent epiblast stem cells from mammalian embryos. Nature.

[bb0040] Buehr M., Meek S., Blair K., Yang J., Ure J., Silva J., McLay R., Hall J., Ying Q.L., Smith A. (2008). Capture of authentic embryonic stem cells from rat blastocysts. Cell.

[bb0045] Canning D.R., Stern C.D. (1988). Changes in the expression of the carbohydrate epitope HNK-1 associated with mesoderm induction in the chick embryo. Development.

[bb0050] Carsience R.S., Clark M.E., Verrinder Gibbins A.M., Etches R.J. (1993). Germline chimeric chickens from dispersed donor blastodermal cells and compromised recipient embryos. Development.

[bb0055] Chambers I., Smith A. (2004). Self renewal of teratocarcinoma and embryonic stem cells. Oncogene.

[bb0060] Chenoweth J.G., McKay R.D., Tesar P.J. (2010). Epiblast stem cells contribute new insight into pluripotency and gastrulation. Dev. Growth Differ..

[bb0065] De Los Angeles A., Loh Y.H., Tesar P.J., Daley G.Q. (2012). Accessing naïve human pluripotency. Curr. Opin. Genet. Dev..

[bb0070] Edson M.A., Lin Y.N., Matzuk M.M. (2010). Deletion of the novel oocyte-enriched gene, Gpr149, leads to increased fertility in mice. Endocrinology.

[bb0075] Evans M.J., Kaufman M.H. (1981). Establishment in culture of pluripotential cells from mouse embryos. Nature.

[bb0080] Eyal-Giladi H., Kochav S. (1976). From cleavage to primitive streak formation: a complementary normal table and a new look at the first stages of the development of the chick. I. General morphology. Dev. Biol..

[bb0085] Gandrillon O., Jurdic P., Benchaibi M., Xiao J.H., Ghysdael J., Samarut J. (1987). Expression of the v-erbA oncogene in chicken embryo fibroblasts stimulates their proliferation in vitro and enhances tumor growth in vivo. Cell.

[bb0090] Gillich A., Bao S., Grabole N., Hayashi K., Trotter M.W., Pasque V., Magnúsdóttir E., Surani M.A. (2012). Epiblast stem cell-based system reveals reprogramming synergy of germline factors. Cell Stem Cell.

[bb0095] Gómez M.C., Serrano M.A., Pope C.E., Jenkins J.A., Biancardi M.N., López M., Dumas C., Galiguis J., Dresser B.L. (2010). Derivation of cat embryonic stem-like cells from in vitro-produced blastocysts on homologous and heterologous feeder cells. Theriogenology.

[bb0100] Guo G., Smith A. (2010). A genome-wide screen in EpiSCs identifies Nr5a nuclear receptors as potent inducers of ground state pluripotency. Development.

[bb0105] Guo G., Yang J., Nichols J., Hall J.S., Eyres I., Mansfield W., Smith A. (2009). Klf4 reverts developmentally programmed restriction of ground state pluripotency. Development.

[bb0110] Ha J.Y., Park T.S., Hong Y.H., Jeong D.K., Kim J.N., Kim K.D., Lim J.M. (2002). Production of germline chimeras by transfer of chicken gonadal primordial germ cells maintained in vitro for an extended period. Theriogenology.

[bb0115] Hamburger V., Hamilton H.L. (1951). A series of normal stages in the development of the chick embryo. J. Morphol..

[bb0120] Han D.W., Tapia N., Joo J.Y., Greber B., Araúzo-Bravo M.J., Bernemann C., Ko K., Wu G., Stehling M., Do J.T., Schöler H.R. (2010). Epiblast stem cell subpopulations represent mouse embryos of distinct pregastrulation stages. Cell.

[bb0125] Hatoya S., Torii R., Kondo Y., Okuno T., Kobayashi K., Wijewardana V., Kawate N., Tamada H., Sawada T., Kumagai D., Sugiura K., Inaba T. (2006). Isolation and characterization of embryonic stem-like cells from canine blastocysts. Mol. Reprod. Dev..

[bb0130] Hayashi K., Surani M.A. (2009). Self-renewing epiblast stem cells exhibit continual delineation of germ cells with epigenetic reprogramming in vitro. Development.

[bb0135] Heider A., Alt R. (2013). virtualArray: a R/bioconductor package to merge raw data from different microarray platforms. BMC Bioinformatics.

[bb0140] Hong N., Li Z., Hong Y. (2011). Fish stem cell cultures. Int. J. Biol. Sci..

[bb0145] Hu J., Wang F., Zhu X., Yuan Y., Ding M., Gao S. (2010). Mouse ZAR1-like (XM_359149) colocalizes with mRNA processing components and its dominant-negative mutant caused two-cell-stage embryonic arrest. Dev. Dyn..

[bb0150] Ikawa M., Tokuhiro K., Yamaguchi R., Benham A.M., Tamura T., Wada I., Satouh Y., Inoue N., Okabe M. (2011). Calsperin is a testis-specific chaperone required for sperm fertility. J. Biol. Chem..

[bb0155] Intarapat S., Stern C.D. (2013). Sexually dimorphic and sex-independent left-right asymmetries in chicken embryonic gonads. PLoS One.

[bb0160] Intarapat S., Stern C.D. (2013). Chick stem cells: current progress and future prospects. Stem Cell Res..

[bb0165] Kumar De A., Malakar D., Akshey Y.S., Jena M.K., Dutta R. (2011). Isolation and characterization of embryonic stem cell-like cells from in vitro produced goat (Capra hircus) embryos. Anim. Biotechnol..

[bb0185] Lavial F., Pain B. (2010). Chicken embryonic stem cells as a non-mammalian embryonic stem cell model. Dev. Growth Differ..

[bb0180] Lavial F., Acloque H., Bertocchini F., Macleod D.J., Boast S., Bachelard E., Montillet G., Thenot S., Sang H.M., Stern C.D., Samarut J., Pain B. (2007). The Oct4 homologue PouV and Nanog regulate pluripotency in chicken embryonic stem cells. Development.

[bb0175] Lavial F., Acloque H., Bachelard E., Nieto M.A., Samarut J., Pain B. (2009). Ectopic expression of Cvh (Chicken Vasa homologue) mediates the reprogramming of chicken embryonic stem cells to a germ cell fate. Dev. Biol..

[bb0190] Li M., Li Y.H., Hou Y., Sun X.F., Sun Q., Wang W.H. (2004). Isolation and culture of pluripotent cells from in vitro produced porcine embryos. Zygote.

[bb0195] Li P., Tong C., Mehrian-Shai R., Jia L., Wu N., Yan Y., Maxson R.E., Schulze E.N., Song H., Hsieh C.L., Pera M.F., Ying Q.L. (2008). Germline competent embryonic stem cells derived from rat blastocysts. Cell.

[bb0200] Macdonald J., Glover J.D., Taylor L., Sang H.M., McGrew M.J. (2010). Characterisation and germline transmission of cultured avian primordial germ cells. PLoS One.

[bb0205] Macdonald J., Taylor L., Sherman A., Kawakami K., Takahashi Y., Sang H.M., McGrew M.J. (2012). Efficient genetic modification and germ-line transmission of primordial germ cells using piggyBac and Tol2 transposons. Proc. Natl. Acad. Sci. U. S. A..

[bb0210] Marks H., Kalkan T., Menafra R., Denissov S., Jones K., Hofemeister H., Nichols J., Kranz A., Stewart A.F., Smith A., Stunnenberg H.G. (2012). The transcriptional and epigenomic foundations of ground state pluripotency. Cell.

[bb0215] Martin G.R. (1981). Isolation of a pluripotent cell line from early mouse embryos cultured in medium conditioned by terato-carcinoma stem cells. Proc. Natl. Acad. Sci. U. S. A..

[bb0220] Masui S., Nakatake Y., Toyooka Y., Shimosato D., Yagi R., Takahashi K., Okochi H., Okuda A., Matoba R., Sharov A.A., Ko M.S., Niwa H. (2007). Pluripotency governed by Sox2 via regulation of Oct3/4 expression in mouse embryonic stem cells.

[bb0225] Michailidis G., Argiriou A., Avdi M. (2010). Expression of chicken zygote arrest 1 (Zar1) and Zar1-like genes during sexual maturation and embryogenesis. Vet. Res. Commun..

[bb0230] Naito M., Sano A., Harumi T., Matsubara Y., Kuwana T. (2004). Migration of primordial germ cells isolated from embryonic blood into the gonads after transfer to stage X blastoderms and detection of germline chimaerism by PCR. Br. Poult. Sci..

[bb0235] Ng H.H., Surani M.A. (2011). The transcriptional and signalling networks of pluripotency. Nat. Cell Biol..

[bb0240] Nichols J., Silva J., Roode M., Smith A. (2009). Suppression of Erk signalling promotes ground state pluripotency in the mouse embryo. Development.

[bb0245] Pain B., Clark M.E., Shen M., Nakazawa H., Sakurai M., Samarut J., Etches R.J. (1996). Long term in vitro culture and characterisation of avian embryonic stem cells with multiple morphogenetic potentialities. Development.

[bb0250] Park T.S., Han J.Y. (2012). piggyBac transposition into primordial germ cells is an efficient tool for transgenesis in chickens. Proc. Natl. Acad. Sci. U. S. A..

[bb0255] Park T.S., Jeong D.K., Kim J.N., Song G.H., Hong Y.H., Lim J.M., Han J.Y. (2003). Improved germline transmission in chicken chimeras produced by transplantation of gonadal primordial germ cells into recipient embryos. Biol. Reprod..

[bb0260] Pashai N., Hao H., All A., Gupta S., Chaerkady R., De Los Angeles A., Gearhart J.D., Kerr C.L. (2012). Genome-wide profiling of pluripotent cells reveals a unique molecular signature of human embryonic germ cells. PLoS One.

[bb0265] Petitte J.N., Liu G., Yang Z. (2004). Avian pluripotent stem cells. Mech. Dev..

[bb9000] Rengaraj D., Lee S.I., Yoo M., Kim T.H., Song G., Han J.Y. (2012). Expression and knockdown analysis of glucose phosphate isomerase in chicken primordial germ cells. Biol Reprod..

[bb0270] Rex M., Orme A., Uwanogho D., Tointon K., Wigmore P.M., Sharpe P.T., Scotting P.J. (1997). Dynamic expression of chicken Sox2 and Sox3 genes in ectoderm induced to form neural tissue. Dev. Dyn..

[bb0275] Schusser B., Collarini E.J., Yi H., Izquierdo S.M., Fesler J., Pedersen D., Klasing K.C., Kaspers B., Harriman W.D., van de Lavoir M.C., Etches R.J., Leighton P.A. (2013). Immunoglobulin knockout chickens via efficient homologous recombination in primordial germ cells. Proc. Natl. Acad. Sci. U. S. A..

[bb0280] Solari F., Flamant F., Cherel Y., Wyers M., Jurdic P. (1996). The osteoclast generation: an in vitro and in vivo study with a genetically labelled avian monocytic cell line. J. Cell Sci..

[bb0285] Song Y., Duraisamy S., Ali J., Kizhakkayil J., Jacob V.D., Mohammed M.A., Eltigani M.A., Amisetty S., Shukla M.K., Etches R.J., de Lavoir M.C. (2014). Characteristics of long-term cultures of avian primordial germ cells and gonocytes. Biol. Reprod..

[bb0290] Stern C.D., Canning D.R. (1990). Origin of cells giving rise to mesoderm and endoderm in chick embryo. Nature.

[bb0300] Streit A., Stern C.D. (2001). Combined whole-mount in situ hybridization and immunohistochemistry in avian embryos. Methods.

[bb0295] Streit A., Berliner A.J., Papanayotou C., Sirulnik A., Stern C.D. (2000). Initiation of neural induction by FGF signalling before gastrulation. Nature.

[bb0305] Tesar P.J., Chenoweth J.G., Brook F.A., Davies T.J., Evans E.P., Mack D.L., Gardner R.L., McKay R.D. (2007). New cell lines from mouse epiblast share defining features with human embryonic stem cells. Nature.

[bb0315] Thomson J.A., Kalishman J., Golos T.G., Durning M., Harris C.P., Becker R.A., Hearn J.P. (1995). Isolation of a primate embryonic stem cell line. Proc. Natl. Acad. Sci. U. S. A..

[bb0310] Thomson J.A., Itskovitz-Eldor J., Shapiro S.S., Waknitz M.A., Swiergiel J.J., Marshall V.S., Jones J.M. (1998). Embryonic stem cell lines derived from human blastocysts. Science.

[bb0320] Toyooka Y., Shimosato D., Murakami K., Takahashi K., Niwa H. (2008). Identification and characterization of subpopulations in undifferentiated ES cell culture. Development.

[bb8000] Tsunekawa N., Naito M., Sakai Y., Nishida T., Noce T. (2000). Isolation of chicken vasa homolog gene and tracing the origin of primordial germ cells. Development.

[bb0325] Urven L.E., Erickson C.A., Abbott U.K., McCarrey J.R. (1988). Analysis of germ line development in the chick embryo using an anti-mouse EC cell antibody. Development.

[bb0330] Uwanogho D., Rex M., Cartwright E.J., Pearl G., Healy C., Scotting P.J., Sharpe P.T. (1995). Embryonic expression of the chicken Sox2, Sox3 and Sox11 genes suggests an interactive role in neuronal development. Mech. Dev..

[bb0335] van de Lavoir M.C., Diamond J.H., Leighton P.A., Mather-Love C., Heyer B.S., Bradshaw R., Kerchner A., Hooi L.T., Gessaro T.M., Swanberg S.E., Delany M.E., Etches R.J. (2006). Germline transmission of genetically modified primordial germ cells. Nature.

[bb0340] van de Lavoir M.C., Mather-Love C., Leighton P., Diamond J.H., Heyer B.S., Roberts R., Zhu L., Winters-Digiacinto P., Kerchner A., Gessaro T., Swanberg S., Delany M.E., Etches R.J. (2006). High-grade transgenic somatic chimeras from chicken embryonic stem cells. Mech. Dev..

[bb0345] Verma V., Gautam S.K., Singh B., Manik R.S., Palta P., Singla S.K., Goswami S.L., Chauhan M.S. (2007). Isolation and characterization of embryonic stem cell-like cells from in vitro-produced buffalo (Bubalus bubalis) embryos. Mol. Reprod. Dev..

[bb0350] Wakamatsu Y., Ozato K., Sasado T. (1994). Establishment of a pluripotent cell line derived from a medaka (Oryzias latipes) blastula embryo. Mol. Mar. Biol. Biotechnol..

[bb0360] West J.A., Viswanathan S.R., Yabuuchi A., Cunniff K., Takeuchi A., Park I.H., Sero J.E., Zhu H., Perez-Atayde A., Frazier A.L., Surani M.A., Daley G.Q. (2009). A role for Lin28 in primordial germ-cell development and germ-cell malignancy. Nature.

[bb0365] Wood H.B., Episkopou V. (1999). Comparative expression of the mouse Sox1, Sox2 and Sox3 genes from pre-gastrulation to early somite stages. Mech. Dev..

[bb0370] Yang J., Gao C., Chai L., Ma Y. (2010). A novel SALL4/OCT4 transcriptional feedback network for pluripotency of embryonic stem cells. PLoS One.

[bb0375] Yi M., Hong N., Hong Y. (2009). Generation of medaka fish haploid embryonic stem cells. Science.

[bb0380] Yoshimura T., Toyoda S., Kuramochi-Miyagawa S., Miyazaki T., Miyazaki S., Tashiro F., Yamato E., Nakano T., Miyazaki J. (2009). Gtsf1/Cue110, a gene encoding a protein with two copies of a CHHC Zn-finger motif, is involved in spermatogenesis and retrotransposon suppression in murine testes. Dev. Biol..

[bb0385] Zhang J., Tam W.L., Tong G.Q., Wu Q., Chan H.Y., Soh B.S., Lou Y., Yang J., Ma Y., Chai L., Ng H.H., Lufkin T., Robson P., Lim B. (2006). Sall4 modulates embryonic stem cell pluripotency and early embryonic development by the transcriptional regulation of Pou5f1. Nat. Cell Biol..

[bb0390] Zhou H., Li W., Zhu S., Joo J.Y., Do J.T., Xiong W., Kim J.B., Zhang K., Schöler H.R., Ding S. (2010). Conversion of mouse epiblast stem cells to an earlier pluripotency state by small molecules. J. Biol. Chem..

