# Supplementary material for: Transcriptome analysis of chicken ES, blastodermal and germ cells reveals that chick ES cells are equivalent to mouse ES cells rather than EpiSC
Source: Stem Cell Res. 2015 Jan;14(1):54–67. doi: 10.1016/j.scr.2014.11.005 (PMC4305369; doi:10.1016/j.scr.2014.11.005)
Supplement: Supplementary file 4 — Fig. S1. Venn diagram of the differentially expressed genes. The Venn diagram presents the genes that are differentially expressed between the tested cell types, including as a reference the Chicken Embryonic Fibroblasts (CEF) (S1A), the monocytic BM2 progenitor cell (S1B), Primordial germ cells (PGC) (S1C) and Chicken blastodermal cells (cBC) from stage X (EG & K) chick embryos (S1D). Fig. 1 illustrates the Venn diagram by taking the Chicken Embryonic Stem (cES) cells as a reference. Fig. S2. The PGC score. The PGC score illustrates the differentially expressed between the PGCs and all the other cell types. This Score is obtained by summing the different Log Fold Change (FC) directly get from the microarray analysis. The higher the score, the more specific are the differentially expressed genes for the cell type. Some genes are listed all along the curve and can be found on Table S2-PGC. The PGC score illustrates the differentially expressed between the PGCs and all the other cell types. This Score is obtained by summing the different Log Fold Change (FC) directly get from the microarray analysis. The higher the score, the more specific are the differentially expressed genes for the cell type. Some genes are listed all along the curve and can be found on Table S2-PGC. Fig. S3. Few tested genes are expressed in the different tested stem cell subtypes. The expression of HEMGN, GPR149, FBXO5, SOCS1 and GPR86 genes was analysed by real time RT-PCR in CEF, BM2, cES, PGC and cBC. Their expression is taken at 1 in cES. These genes are the only ones among those tested in which a significant expression was detected in BM2 cells. SOCS1 is also slightly expressed in CEF. Each sample was run in triplicates. [file mmc4.pptx]

## Slide 1
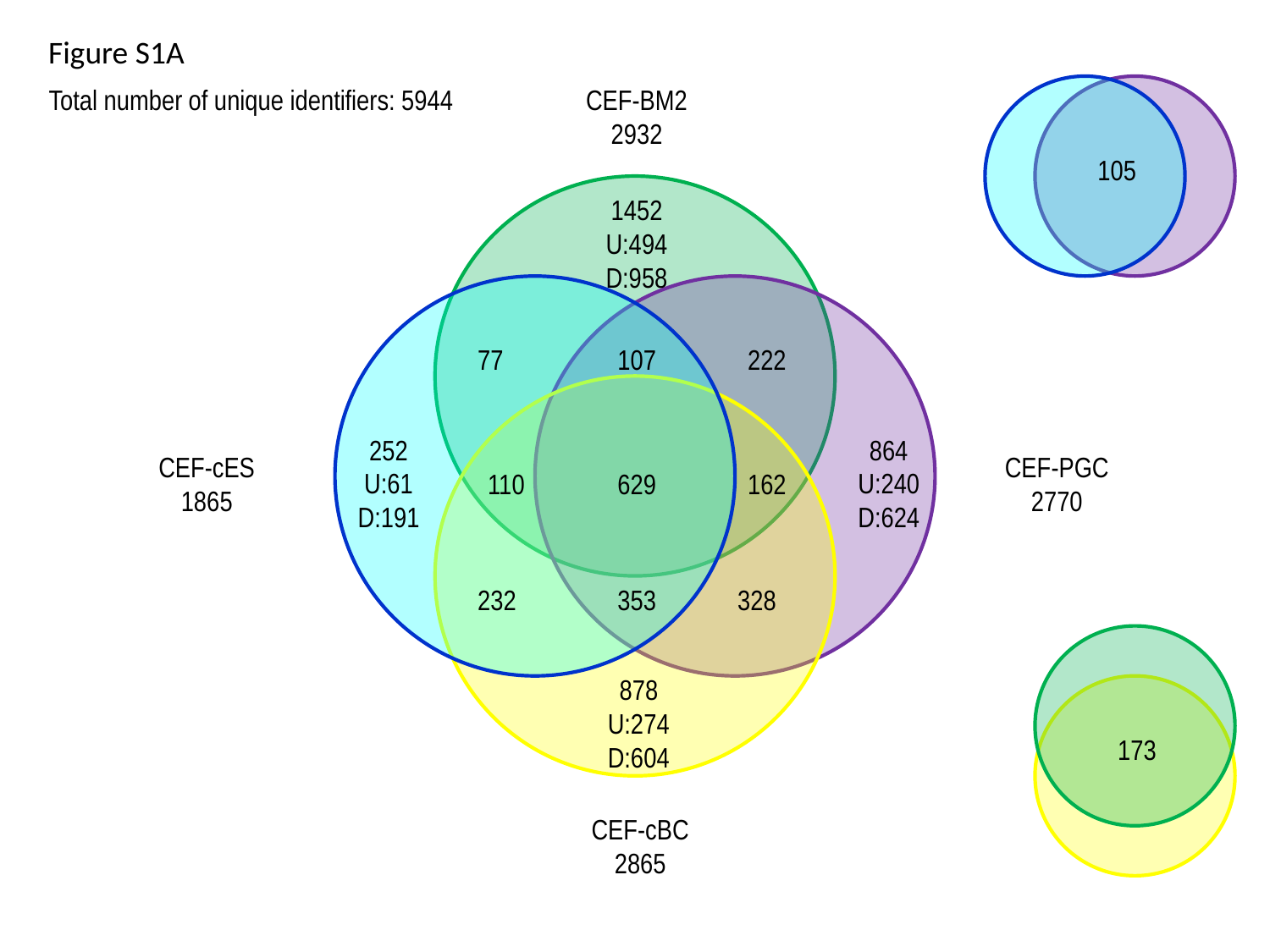

Figure S1A
Total number of unique identifiers: 5944
CEF-BM2
2932
105
1452
U:494
D:958
77
107
222
252
U:61
D:191
864
U:240
D:624
CEF-cES
1865
CEF-PGC
2770
110
629
162
232
353
328
878
U:274
D:604
173
CEF-cBC
2865

## Slide 2
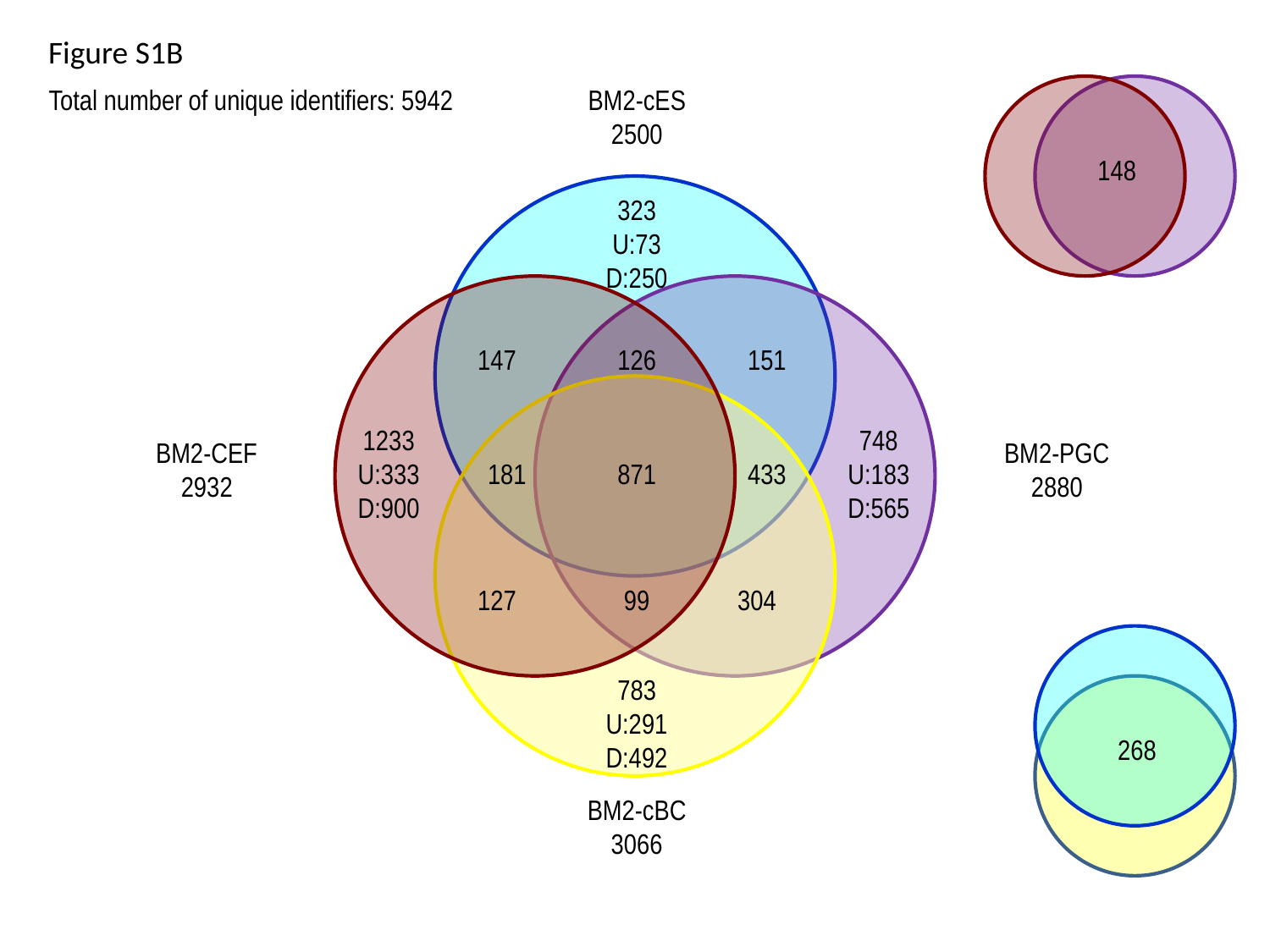

Figure S1B
Total number of unique identifiers: 5942
BM2-cES
2500
148
323
U:73
D:250
147
126
151
1233
U:333
D:900
748
U:183
D:565
BM2-CEF
2932
BM2-PGC
2880
181
871
433
127
99
304
783
U:291
D:492
268
BM2-cBC
3066

## Slide 3
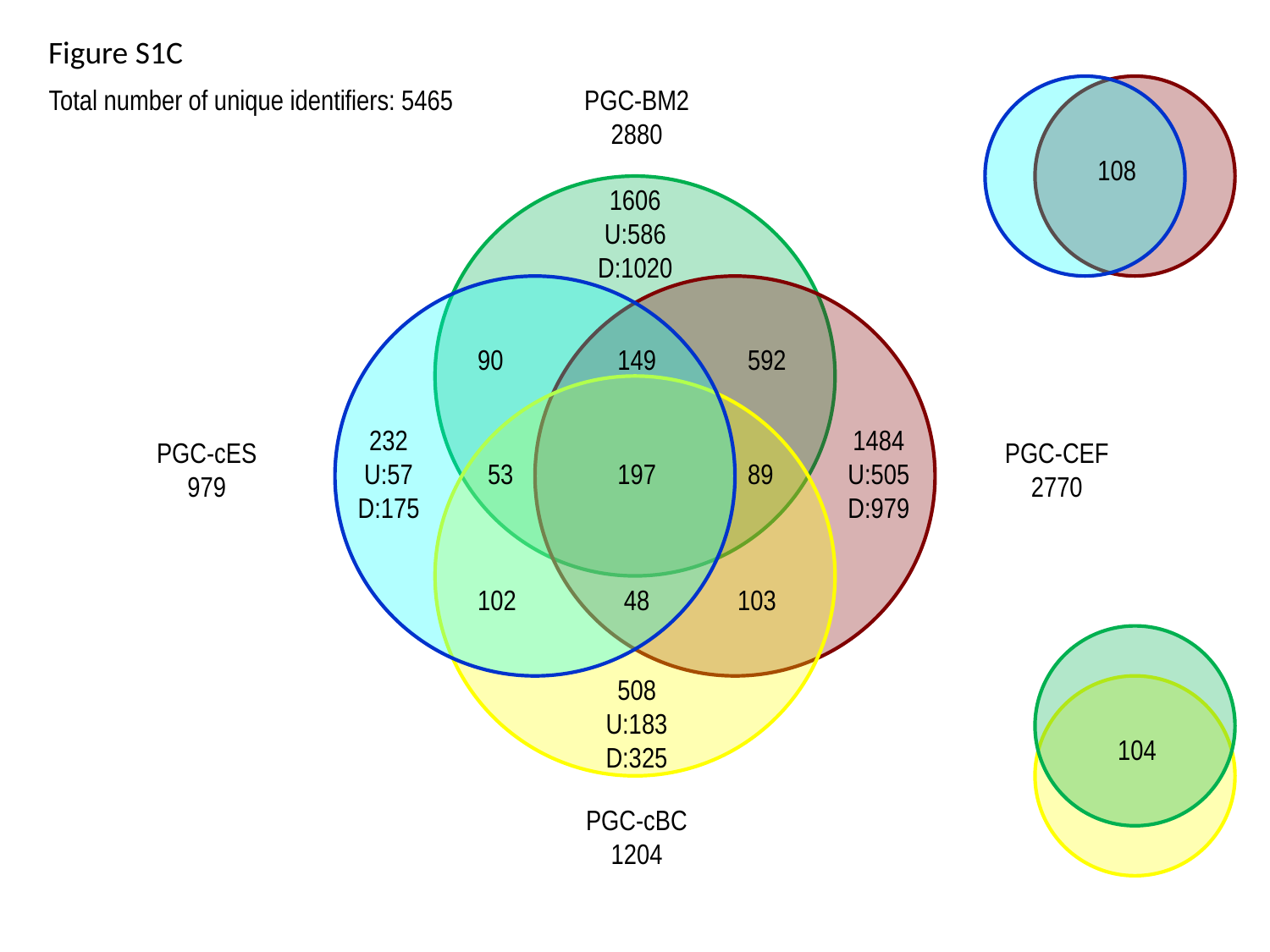

Figure S1C
Total number of unique identifiers: 5465
PGC-BM2
2880
108
1606
U:586
D:1020
90
149
592
232
U:57
D:175
1484
U:505
D:979
PGC-cES
979
PGC-CEF
2770
53
197
89
102
48
103
508
U:183
D:325
104
PGC-cBC
1204

## Slide 4
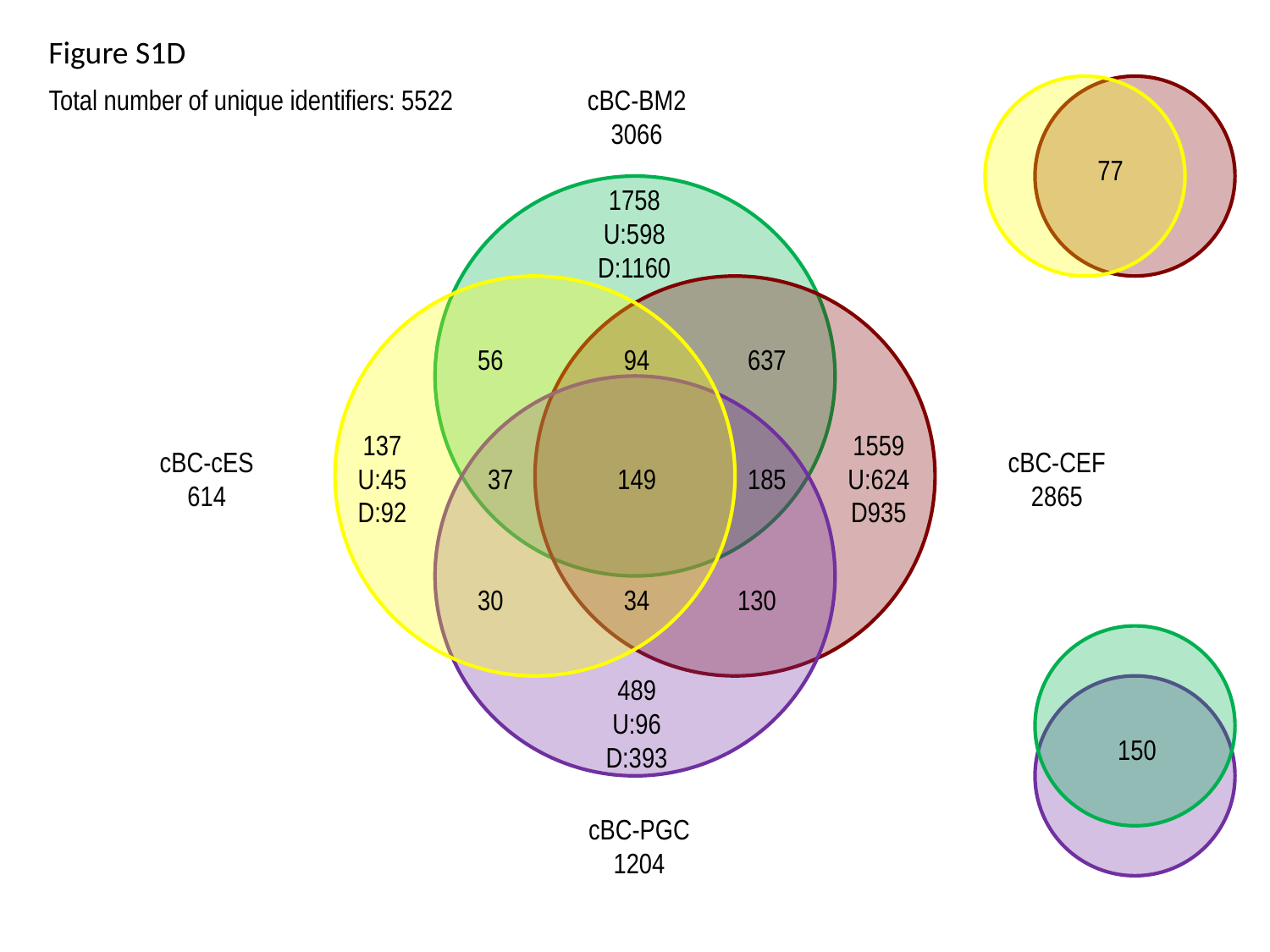

Figure S1D
Total number of unique identifiers: 5522
cBC-BM2
3066
77
1758
U:598
D:1160
56
94
637
137
U:45
D:92
1559
U:624
D935
cBC-cES
614
cBC-CEF
2865
37
149
185
30
34
130
489
U:96
D:393
150
cBC-PGC
1204

## Slide 5
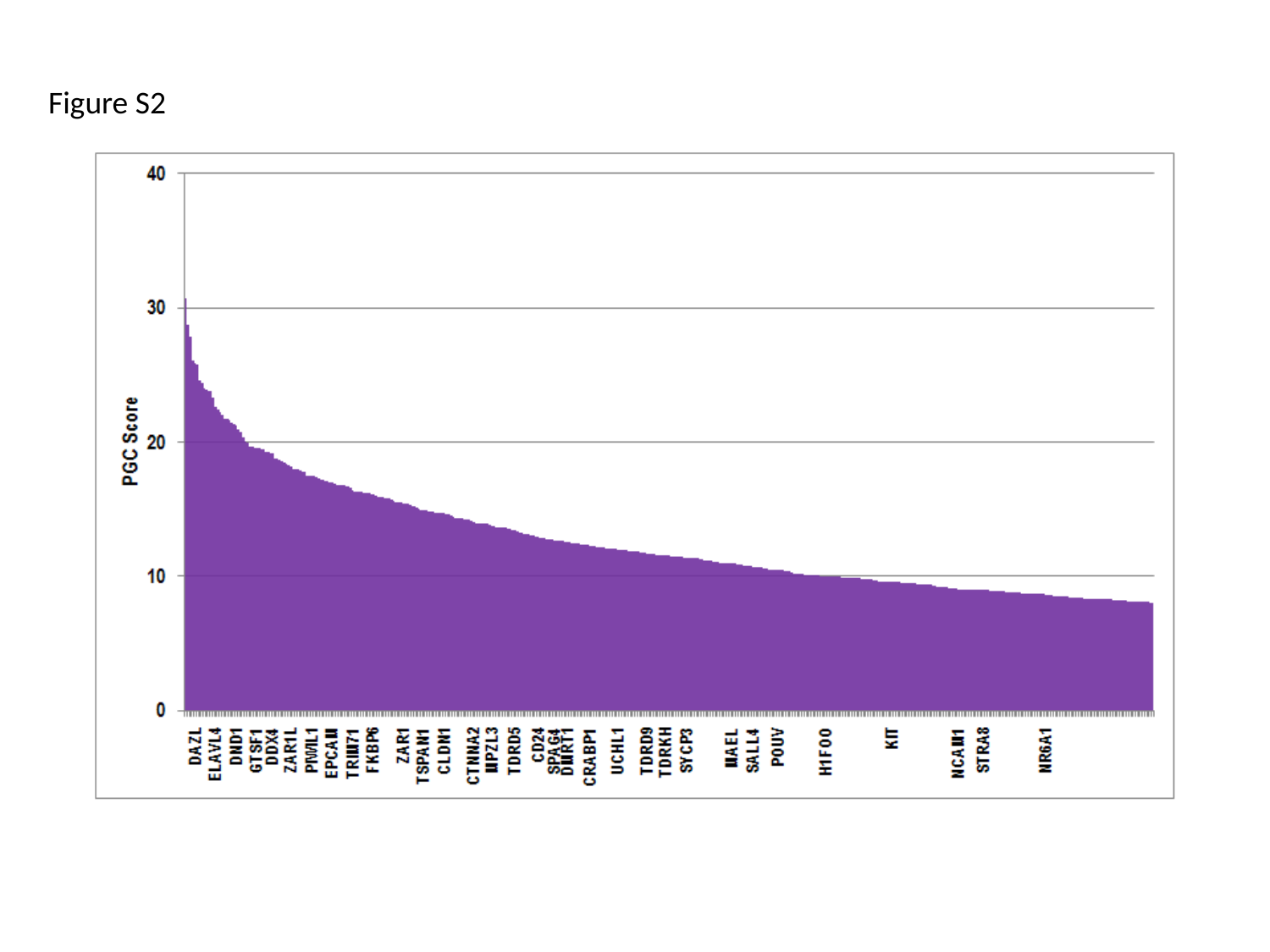

Figure S2

## Slide 6
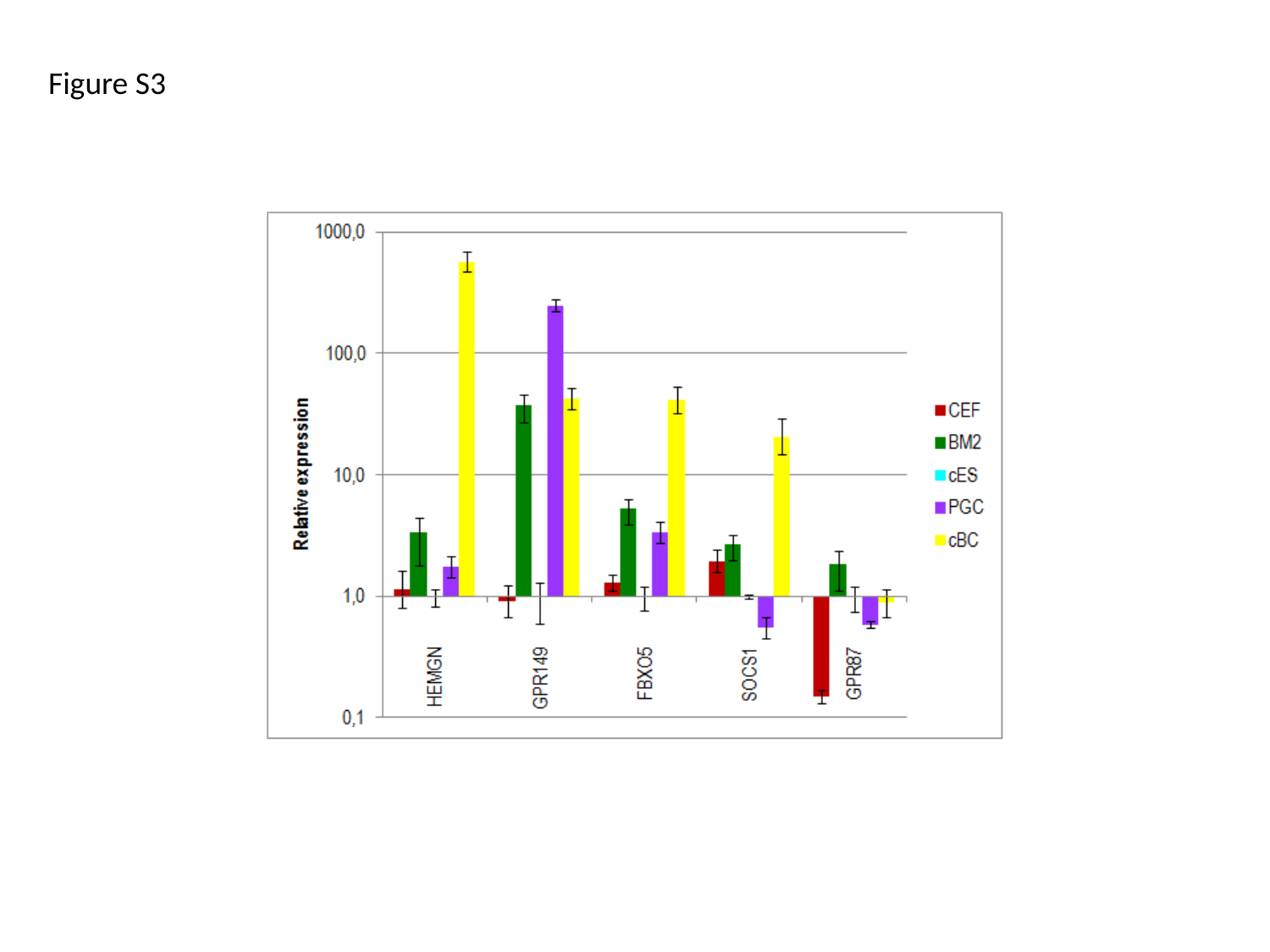

Figure S3
